# Exogenous AMPA downregulates gamma-frequency network oscillation in CA3 of rat hippocampal slices

**DOI:** 10.1038/s41598-023-36876-w

**Published:** 2023-06-29

**Authors:** Chengzhang Li, Zhenrong Li, Sihan Xu, Sanwei Jiang, Zhenli Ye, Bin Yu, Shixiang Gong, Junmei Li, Qilin Hu, Bingyan Feng, Mengmeng Wang, Chengbiao Lu

**Affiliations:** 1 Henan International Key Laboratory for Noninvasive Neuromodulation/Key Laboratory of Brain Research of Henan Province, Department of Physiology & Pathophysiology, School of Basic Medical Science, Xinxiang Medical University, Xinxiang, China; 2 School of Information Science and Engineering, Yanshan University, Qinhuangdao, China

**Keywords:** Neuroscience, Physiology

## Abstract

Pharmacologically-induced persistent hippocampal γ oscillation in area CA3 requires activation of α-Amino-3-hydroxy-5-methyl-4-isoxazolepropionate receptors (AMPARs). However, we demonstrated that exogenous AMPA dose-dependently inhibited carbachol (CCH)-induced γ oscillation in the CA3 area of rat hippocampal slices, but the underlying mechanism is not clear. Application of AMPARs antagonist NBQX (1 μM) did not affect γ oscillation power (γ power), nor AMPA-mediated γ power reduction. At 3 μM, NBQX had no effect on γ power but largely blocked AMPA-mediated γ power reduction. Ca^2+^-permeable AMPA receptor (CP-AMPAR) antagonist IEM1460 or CaMKK inhibitor STO-609 but not CaMKIIα inhibitor KN93 enhanced γ power, indicating that activation of CP-AMPAR or CaMKK negatively modulated CCH-induced γ oscillation. Either CP-AMPAR antagonist or CaMKK inhibitor alone did not affected AMPA-mediated γ power reduction, but co-administration of IEM1460 and NBQX (1 μM) largely prevented AMPA-mediated downregulation of γ suggesting that CP-AMPARs and CI-AMPARs are involved in AMPA downregulation of γ oscillation. The recurrent excitation recorded at CA3 stratum pyramidale was significantly reduced by AMPA application. Our results indicate that AMPA downregulation of γ oscillation may be related to the reduced recurrent excitation within CA3 local neuronal network due to rapid CI-AMPAR and CP-AMPAR activation.

## Introduction

The brain neural networks are prone to generate oscillations due to the intrinsic connectivity properties^1,2^. Neural oscillations at γ band (30–80 Hz, γ oscillation) are present in multiple brain areas including hippocampus, thalamus and neocortex and associated with higher brain functions such as sensory processing, learning and memory^3–7^. Generated from interactions between inhibitory interneurons and excitatory pyramidal cells^8^, γ oscillation provides precise time coding for the firing of neurons within the network and regulates synaptic signal transmission and plasticity^9–11^. Aberrant γ oscillations have been linked to cognitive disorders such as Alzheimer’s disease (AD) and schizophrenia^12–14^. Recent rodent and human studies provided evidence showing that γ-band stimulation exert neuroprotective effect in age- and AD-related brain disorders^15^. 40 Hz γ wave stimulation reduced amyloid beta toxicity and improved cognitive functions in animal models of AD^16,17^. Human study indicated that γ oscillations in cortical motor areas contribute to plasticity processes and enhancing γ oscillations restores plasticity in Parkinson's Disease in primary motor cortex^18^. Pharmacologically-induced γ oscillation in the CA3 subfield of hippocampus may last for hours^19^ and thus has been widely used as an in vitro model for the study of the mechanism and modulation of γoscillation^20^. 

α-amino-3-hydroxy-5-methyl-4-isoxazole- propionate receptors (AMPARs), the glutamate ionotropic receptors^21,22^ are tetrameric ligand-gated channels composed of four subunits (GluA1-4)^23^. AMPARs play a major role in the maintenance of γ oscillation^24^. The AMPARs with GluA2 subunit are Ca^2+^-impermeable AMPA receptors (CI-AMPARs) and those lack of GluA2 are Ca^2+^-permeable AMPA receptors (CP-AMPARs)^25^. Ca^2+^ inflow through CP-AMPAR triggers Ca^2+^ release from internal store, which is a main mechanism of Ca^2+^ signaling and synaptic function in fast spiking interneurons^26,27^. Ca^2+^ influx activates calmodulin-dependent protein kinase kinase (CaMKK) and its downstream signaling molecule calcium/calmodulin dependent protein kinase II (CaMKII), thereby affecting synaptic plasticity via regulation of synaptic expression of CP-AMPAR^28–31^.

It was reported that CCH-induced γ oscillation requires AMPAR activation^32,33^. However, our study showed the exogenous AMPA application rapidly suppressed CCH-induced γ oscillation and explored the mechanisms for AMPA modulation of γ oscillation.

## Results

### The effect of AMPAR agonist AMPA on rat hippocampal γ oscillations

**Figure 1:**
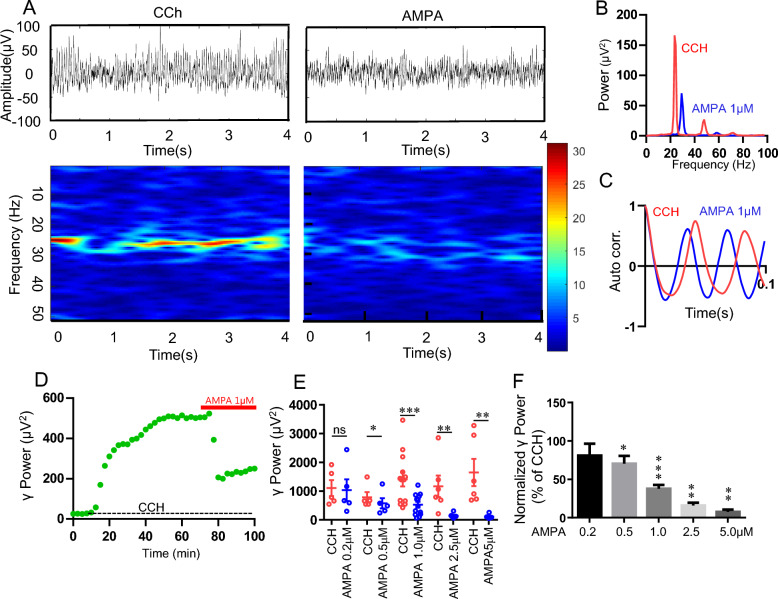
The effect of exogenous AMPA on CCH-induced γ oscillations in CA3 area of rat hippocampal slices. (**A**) Example raw traces (top panels) and time–frequency plot (low panel) of field potentials recorded in hippocampal CA3 area in the presence of CCh and CCH + AMPA (1 μM). (**B**) The power spectra of CCH-induced γ oscillations before and after application of AMPA. (**C**) Corresponding autocorrelograms of the oscillations showed in (**A**). (**D**) The representative time-effect curve of hippocampal γ power before and after application of AMPA (1 μM). The field potential was recorded from the hippocampal CA3 area of a male SD rat slice. (**E**) Scatter plots of γ power for AMPA at different concentration (0.2–5.0 μM). The red empty circles represent γ power for CCH alone, and the blue ones represent γ power for different concentrations of AMPA in presence of CCH. (**F**) The γ power normalized to the CCH for different concentrations of AMPA (0.2–5.0 μM).

To evaluate the effect of AMPA on hippocampal γ oscillation, CCH (5 μM) was applied to hippocampal slices to induce persistent γ oscillations, followed by perfusion of various concentrations of AMPA (0.2–5 μM). AMPA (1 μM) caused a rapid decrease in γ oscillation power (γ power) (Fig. 1A,B) and a significant increase in peak frequency (Fig. 1B). The autocorrelation graphs of the field potentials confirmed that AMPA treatment did not alter the synchrony of γ oscillations, but increased the peak frequency (Fig. 1C). The time-effect curve before and after application of AMPA (Fig. 1D) showed that AMPA induced a rapid downregulation of γ power, which was maintained constantly at a low level during 20 min perfusion of AMPA. Figure 1E,F showed that AMPA concentration-dependently inhibited hippocampal γ oscillations. At 0.2 μM, AMPA had no effect on γ power (90 ± 15% of CCH, t_(5)_ = 1.108, *P* = 0.318, n = 6 slices from 4 rats). AMPA caused 30% (70 ± 10% of CCH, t_(4)_ = 3.510, *P* = 0.025, n = 5 slices from 3 rats), 62% (38 ± 5% of CCH, t_(11)_ = 4.721, *P* = 0.001, n = 12 slices from 8 rats) and 84% reduction in γ power (16 ± 4% of CCH, t_(5)_ = 2.831, *P* = 0.037, n = 6 slices from 3 rats) for 0.5 μM, 1 μM and 2.5 μM AMPA, respectively. When the concentration of AMPA reached to 5 μM, γ oscillations was completely degraded (7.6 ± 1% of CCH, t_(5)_ = 3.399, *P* = 0.019, n = 6 slices from 3 rats). Although it decreased γ power, AMPA increased oscillatory peak frequency (CCH 23.9 ± 0.3 Hz vs 0.5 μM AMPA 28 ± 0.7 Hz, t_(4)_ = − 4.123, *P* = 0.026, n = 5 slices from 3 rats; CCH 23.6 ± 0.6 Hz vs 1 μM AMPA 31 ± 2 Hz, t _(11)_ =  − 3.587, *P* = 0.004, n = 12 slices from 8 rats).

We also tested the effect of AMPA on kainite (200 nM) -induced γ oscillation. AMPA (5 μM) dramatically inhibited kainate-induced γ oscillation (10 ± 6% of KA, t_(4)_ = 3.031, *P* = 0.039, n = 5 slices from 3 rats) and increased the peak frequency (27.5 ± 1.4 Hz, vs control 25.8 ± 1.7 Hz, t_(4)_ = − 4.224, *P* = 0.013, n = 5 slices from 3 rats). These results indicate that the role of AMPA on γ oscillation is γ model-independent.

### The effect of AMPAR antagonist NBQX on AMPA modulation of γ oscillation

**Figure 2:**
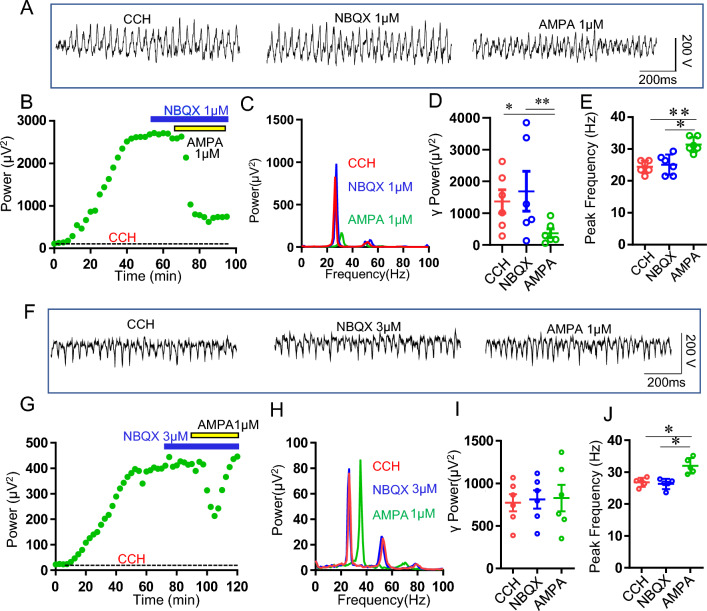
The effect of NBQX on AMPA modulation of γ oscillation. (**A**) Example traces of field potentials recorded in hippocampal CA3 for CCH only, CCH + NBQX (1 μM) and CCH + NBQX (1 μM) + AMPA (1 μM). (**B**) The representative time-effect curve of γ power for CCH alone, CCH + NBQX (1 μM) and CCH + NBQX (1 μM) + AMPA (1 μM). The field potential was recorded from the hippocampal CA3 area of a male SD rat slice. (**C**) Power spectra of the oscillatory activity for CCH alone (red line), CCH + NBQX (blue line) and CCH + NBQX + AMPA (green line) corresponding to the field potentials displayed in (**A**). (**D**) The scatter plots of γ power for CCH only (red circles), CCH + NBQX (blue circles) and CCH + NBQX (1 μM) + AMPA (1 μM) (green circles), respectively. (**E**) The peak frequency of the oscillatory activity for CCH alone, CCH + NBQX (1 μM) and CCH + NBQX (1 μM) + AMPA (1 μM). (**F**) Example traces of field potentials recorded in hippocampal CA3 for CCH only, CCH + NBQX (3 μM) and CCH + NBQX (3 μM) + AMPA (1 μM). (**G**) The representative time-effect curve of γ power for CCH alone, CCH + NBQX (3 μM) and CCH + NBQX (3 μM) + AMPA (1 μM). The field potential was recorded from the hippocampal CA3 area of a male SD rat slice. (**H**) Power spectra of the oscillatory activity for CCH alone (red line), CCH + NBQX (blue line) and CCH + NBQX + AMPA (green line) corresponding to the field potentials displayed in (**F**). (**I**) The scatter plots of γ power for CCH only (red circles), CCH + NBQX (3 μM) (blue circles) and CCH + NBQX (3 μM) + AMPA (1 μM) (green circles), respectively. (**J**) The peak frequency of the oscillatory activity for CCH alone, CCH + NBQX (3 μM) and CCH + NBQX (3 μM) + AMPA (1 μM).

AMPAR antagonist NBQX at 20 μM abolished CCH-induced γ oscillations^34^. We applied a low concentration of NBQX (1 μM) to see whether this concentration is able to block the role of AMPA on γ oscillation. When CCH-induced γ oscillations reached a plateau, NBQX was added into the perfusion solution. No significant change was found in γ power after NBQX application for 20–30 min (118 ± 17% of CCH, vs CCH, t_(5)_ = 0.751, *P* = 0.47, n = 6 slices from 4 rats, Fig. 2A–D). Further application of AMPA (1 μM) caused a rapid, significant reduction in γ power within 5–10 min (34 ± 13% of CCH, vs CCH, *P < 0.05, n = 6 slices from 4 rats) (Fig. 2B) and an increase in the peak frequency (Fig. 2C,E). NBQX did not affect the peak frequency of CCH-induced γ oscillations (25 ± 1.6 Hz vs CCH 24 ± 1.1 Hz, t_(5)_ = 0.398, *P* = 0.648, n = 6 slices from 4 rats) nor AMPA-induced increase in peak frequency (NBQX + AMPA 31.5 ± 1.2 Hz, vs NBQX alone, t_(5)_ = 3.837, *P* = 0.003, n = 6 slices from 4 rats; vs CCH alone, t_(5)_ = 3.512, P = 0.00794, n = 6 slices from 4 rats; vs AMPA alone, t_(16)_ = 0.163, *P* = 0.872, Fig. 2E).

At a concentration of 3 μM, NBQX had no effect on CCH-induced γ oscillation, but affected the dynamics of AMPA modulation of γ oscillation. In the presence of NBQX, AMPA(1 μM) no longer caused a constant suppression of γ oscillations, but sometimes an initial temporary downregulation followed by a rapid recovery of γ power or little inhibition on γ oscillations (99 ± 1% of NBQX, vs NBQX or CCH, *P* > 0.05, n = 6 slices from 3 rats, Fig. 2F–J). These results indicate that AMPA-mediated downregulation of γ oscillation was involved in CI-AMPAR activation.

### The effects of CP-AMPAR antagonist IEM1460, L-type VDCC blocker Nifedipine and NMDAR antagonist D-AP5 on AMPA-mediated reduction of γ oscillation

**Figure 3:**
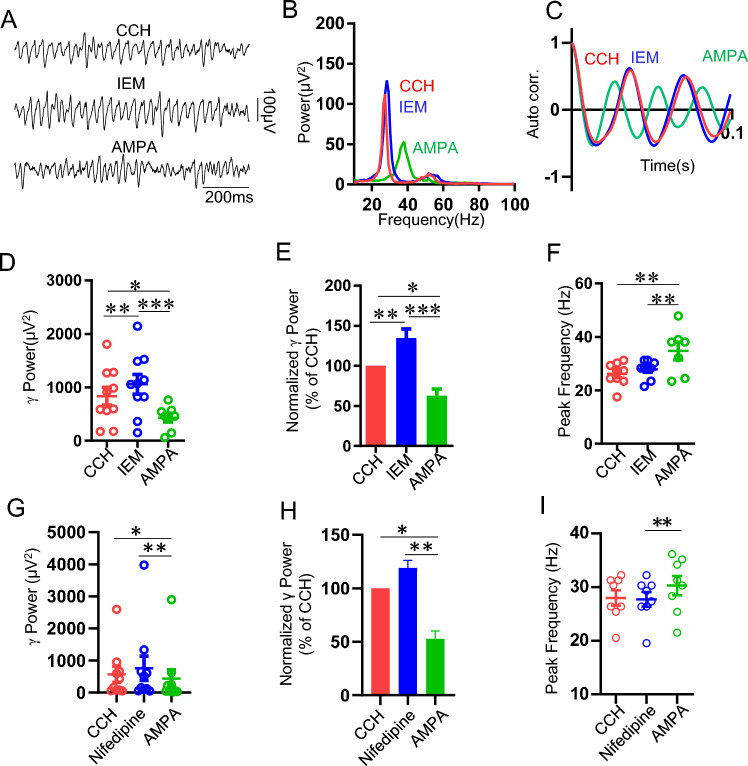
Effect of IEM1460 and nifidepine on AMPA modulation of γ oscillation. (**A**) Example traces of field potentials recorded in hippocampal CA3 for CCH only, CCH + IEM1460 and CCH + IEM1460 + AMPA from a male SD rat. (**B**) Power spectra of the oscillatory activity for CCH alone (red line), CCH + IEM1460 (blue line) and CCH + IEM1460 + AMPA (green line) corresponding to the slice in (**A**). (**C**) Corresponding autocorrelograms of the oscillations displayed in (**A**). (**D**) The scatter plots of γ power for CCH only (red circles), CCH + IEM1460 (blue circles) and CCH + IEM1460 + AMPA (green circles), respectively. (**E**) The γ power normalized to CCH for CCH only, CCH + IEM1460 and CCH + IEM1460 + AMPA. (**F**) The peak frequency of oscillatory activity for CCH, CCH + IEM1460 and CCH + IEM1460 + AMPA. (**G**) The scatter plots of γ power for CCH (red circles), CCH + Nif (blue circles) and CCH + Nif + AMPA (green circles). (**H**) The normalized γ power for CCH only, CCH + Nif and CCH + Nif + AMPA. (**I**) The peak frequency of the oscillatory activity for CCH alone, CCH + Nif and CCH + Nif + AMPA.

To test the role of CP-AMPAR on AMPA-mediated downregulation of γ oscillation, when CCH-induced γ oscillation reached a steady state, application of CP-AMPAR antagonist IEM1460 (10 μM) resulted in significant enhancement of γ power (135 ± 12% vs CCH control, t_(9)_ = − 5.173,** *P* = 0.005, n = 10 slices from 5 rats, Fig. 3A,B,D,E) but had no effect on oscillatory peak frequency (28 ± 1.1 Hz vs CCH control 26 ± 1.4 Hz, t_(8)_ = 1.063, *P* = 0.306, n = 9 slices from 5 rats, Fig. 3F). When the effect of IEM1460 reached to a plateau, further application of AMPA (1 μM) caused a significant reduction in γ power (45 ± 3% of CCH, vs CCH, t_(9)_ = 2.858, *P = 0.0128; vs IEM1460, t_(9)_ = 4.68,*** *P* = 0.00035, n = 10 slices from 5 rats, Fig. 3A,B,D,E) , reduced synchronization (Fig. 3C) but increased oscillatory peak frequency (35 ± 3.2 Hz vs CCH control 26 ± 1.4 Hz, t_(8)_ = 4.67, *P* = 0.000361, n = 9 slices from 5 rats, Fig. 3B,C,F). These results indicate that activation of CP-AMPAR downregulates γ oscillation and blocking CP-AMPAR did not prevent the downregulation of AMPA on γ oscillations.

It was known that L-type calcium channel blocker nifedipine increased hippocampal γ oscillation in aged mice^35^ and calcium channel contributes to the regulation of hippocampal γ oscillations via modulation of intrinsic neuronal excitability^36^. Thus, it is plausible that AMPA-induced down-regulation of γ oscillation may be involved in calcium channel activation. To test this hypothesis, when CCH-induced γ oscillation was stabilized, nifedipine (Nif, 10 μM) application had no effect on CCH-γ power (119 ± 7% of CCH, vs CCH, t_(9)_ = 1.784, *P* = 0.0974, n = 10 slices from 5 rats, Fig. 3G,H), nor on AMPA-induced downregulation of γ power (53 ± 6.8% of CCH, vs CCH, t_(9)_ = 2.976, *P* = 0.00809, n = 10 slices from 5 rats; vs AMPA alone, t_(20)_ = 1.630, *P* = 0.119; Fig. 3G,H). Nif did not alter peak frequency of CCH-induced oscillations (CCH + Nif 28.3 ± 1.3 Hz vs CCH 28.5 ± 1.3 Hz, Fig. 2I), nor on AMPA-mediated increase in peak frequency (CCH + Nif + AMPA 30.3 ± 1.8 Hz vs CCH + Nif 28.5 ± 1.3 Hz; t_(7)_ = 4.089, *P* = 0.00111, Fig. 3I).

The exogenous AMPA may activate CI-AMPAR, causing Na^+^ influx which is strong enough to depolarize cell membrane to activate NMDAR. We then evaluated the effect of NMDA receptor antagonist D-AP5 on AMPA downregulation of CCH-induced γ oscillations. Our results showed that D-AP5 did not affect γ power (124.5 ± 13% of CCH, vs CCH, t = 0.54, *P* = 0.604, n = 5 slices from 2 rats) and nor AMPA-mediated downregulation of γ oscillations (39 ± 5% of D-AP5, vs D-AP5, t_(4)_ = 4.074, *P* = 0.004, n = 5 slices from 2 rats), indicating NMDARs did not involve in CCH-induced γ oscillations nor AMPA-mediated downregulation of γ oscillations.

### The effects of CAMKK inhibitor STO-609 and CAMKII inhibitor KN93 on AMPA-mediated reduction of γ oscillation

The inhibitory neurons express relatively low NMDAR but abundant CP-AMPAR, which is a main source for the dendritic Ca^2+^ signaling of interneurons^36^. CP-AMPAR-mediated Ca^2+^ influx may trigger endoplasmic Ca^2+^ release from ryanodine receptor, which amplify Ca^2+^ signaling and mediate synaptic transmission and plasticity in interneurons^37,38^. Previous study also showed that CP-AMPARs contribute to activity-dependent CaMKII T286 phosphorylation^39^. It is thus possible that activation of CP-AMPAR may affect CaMKK/CaMKII signaling.

**Figure 4:**
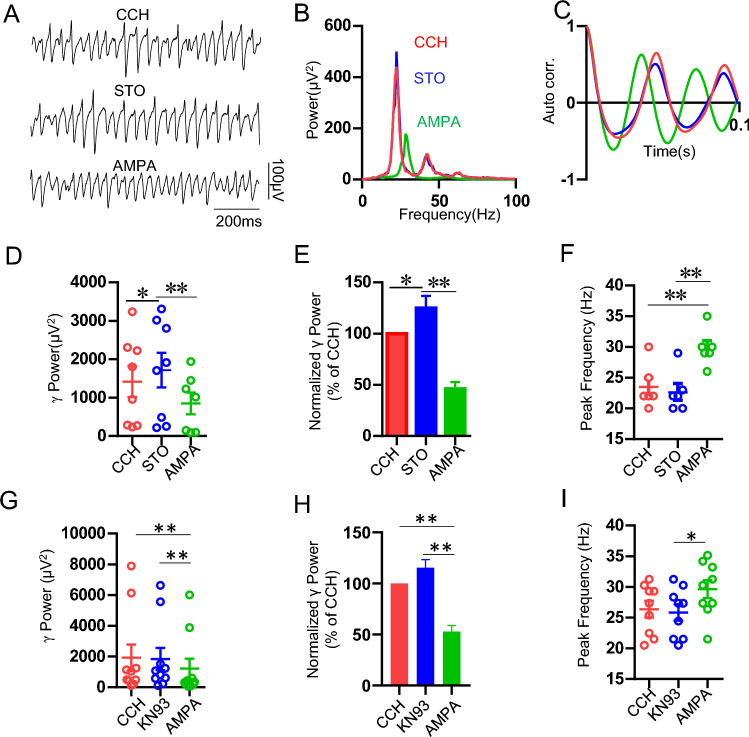
Effect of CAMKK inhibitor STO-609 (10 μM) and CaMKIIα inhibitor KN93 on AMPA modulation of γ oscillation. (**A**) Example traces of field potentials recorded in hippocampal CA3 for CCH only, CCH + STO-609 and CCH + STO-609 + AMPA from a male SD rat. (**B**) Power spectra of the oscillatory activity for CCH alone (red line), CCH + STO-609 (blue line) and CCH + STO-609 + AMPA (green line) corresponding to the slice in (**A**). (**C**) Corresponding autocorrelograms of the oscillations displayed in (**A**). (**D**) The scatter plots of γ power for CCH alone (red circles), CCH + STO-609 (blue circles) and CCH + STO-609 + AMPA (green circles), respectively. (**E**) The γ power normalized to the CCH only baseline value for CCH only (control), CCH + STO-609 and CCH + STO-609 + AMPA. (**F**) The peak frequency of the oscillatory activity for CCH alone, CCH + STO-609 and CCH + STO-609 + AMPA. (**G**) The scatter plots of γ power for CCH alone (red circles), and, CCH + KN93 (blue circles) and CCH + KN93 + AMPA (green circles). (**H**) The normalized γ power for CCH only, CCH + KN93 and CCH + KN93 + AMPA. (**I**) The peak frequency of the oscillatory activity for CCH alone, CCH + KN93 and CCH + KN93 + AMPA.

To determine the role of CAMKK/CaMKIIα in AMPA-mediated downregulation of γ oscillations, CAMKK inhibitor STO-609 (10 μM) was applied for 20 min, which caused a significant increase in γ power (128 ± 9% of CCH, t_(7)_ = − 2.718, *P* = 0.03, n = 8 slices from 4 rats, Fig. 4A–E). AMPA was then applied into perfusion solution for another 20–30 min. The results showed that STO-609 alone enhanced γ oscillation but did not affect AMPA-induced reduction of γ power (46 ± 7% of CCH, vs CCH, t_(7)_ = 2.437, *P* = 0.0299; vs STO, t_(7)_ = 3.66, *P* = 0.00288, n = 8 slices from 4 rats; vs AMPA alone, t_(17)_ = 0.935, *P* = 0.363, Fig. 4A–E). These results indicate that CAMKK activation negatively modulated CCH-induced γ oscillations.

Application of CaMKIIα inhibitor KN93 (10 μM) had no effect on γ oscillations (115 ± 8% of CCH, vs CCH, t_(9)_ = 0.418, *P* = 0.683, n = 10 slices from 4 rats, Fig. 4G,H) nor AMPA downregulation of γ (53 ± 6% of CCH, vs CCH, t_(9)_ = 3.668, *P* = 0.00176; vs KN93, t_(9)_ = 3.25, *P* = 0.00445, n = 10 slices from 4 rats, Fig. 4G,H). Neither STO-609 nor KN93 had any effect on peak frequency nor AMPA-induced increase in peak frequency (Fig. 4F,I). These results indicate that CAMKK/CaMKIIα was not involved in AMPA downregulation of γ oscillation.

### The combined effect of NBQX and IEM1460 on the AMPA-mediated γ oscillation

**Figure 5:**
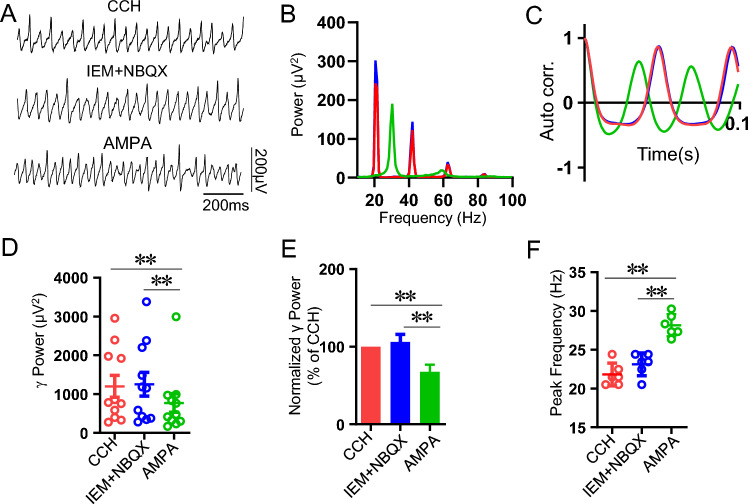
The combined effect of NBQX and IEM1460 on AMPA modulation of γ oscillation. (**A**) Example traces of field potentials recorded in hippocampal CA3 from a male SD rat slice for CCH only, NBQX + IEM1460 and NBQX + IEM1460 + AMPA. (**B**) Power spectra of the oscillatory activity for CCH alone (red line), CCH + NBQX + IEM1460 (blue line) and CCH + NBQX + IEM1460 + AMPA (green line) corresponding to the field potentials shown in (**A**). (**C**) Corresponding autocorrelograms of the oscillations displayed in (**A**). (**D**) The scatter plots of γ power for CCH alone (red circles), NBQX + IEM1460 (blue circles) and NBQX + IEM1460 + AMPA (green circles). (**E**) The γ power normalized to the CCH for baseline (CCH control), NBQX + IEM1460 and NBQX + IEM1460 + AMPA, respectively. (**F**) The peak frequency of the oscillatory activity for CCH alone, NBQX + IEM1460 and NBQX + IEM1460 + AMPA, respectively.

We found that NBQX at 1 μM or IEM1460 at 10 μM alone had no effect on AMPA-mediated γ oscillations. We thus tested the combined effect of NBQX and IEM1460 on AMPA-mediated γ oscillations. When CCH-induced γ oscillation stabilized, combined application of NBQX(1 μM) and IEM1460(10 μM) had no effect on γ power (106 ± 9.6% of CCH, vs CCH, t_(10)_ = 0.383, *P* = 0.706, n = 11 slices from 5 rats, Fig. 5A–E), further application of AMPA(1 μM) caused a 32% decrease in γ power (68 ± 9% of CCH, vs CCH, t_(10)_ = 3.165, *P* = 0.00487, n = 11 slices from 5 rats, Fig. 5A–E). This decrease was smaller than that (62% decrease in γ power) induced by 1 μM AMPA (vs AMPA alone 38 ± 5% of CCH, t_(21)_ = 2.880, *P* = 0.009). These results indicate that the co-administration of NBQX and IEM1460 largely blocked the AMPA-mediated decrease of γ oscillation. Combined application of NBQX + IEM1460 did not affect oscillatory peak frequency (NBQX + IEM1460: 23 ± 0.6 Hz, vs CCH: 22 ± 0.6 Hz, t_(5)_ = 1.727, *P* = 0.115, Fig. 5F) nor AMPA-mediated increase of peak frequency (28 ± 0.5 Hz vs CCH, t_(5)_ = 8. 417, *P* = 0.00000752, Fig. 5F).

### The effect of AMPA on the synaptic transmission of CA3 from mossy fibers and CA3 recurrent collateral fibers

**Figure 6:**
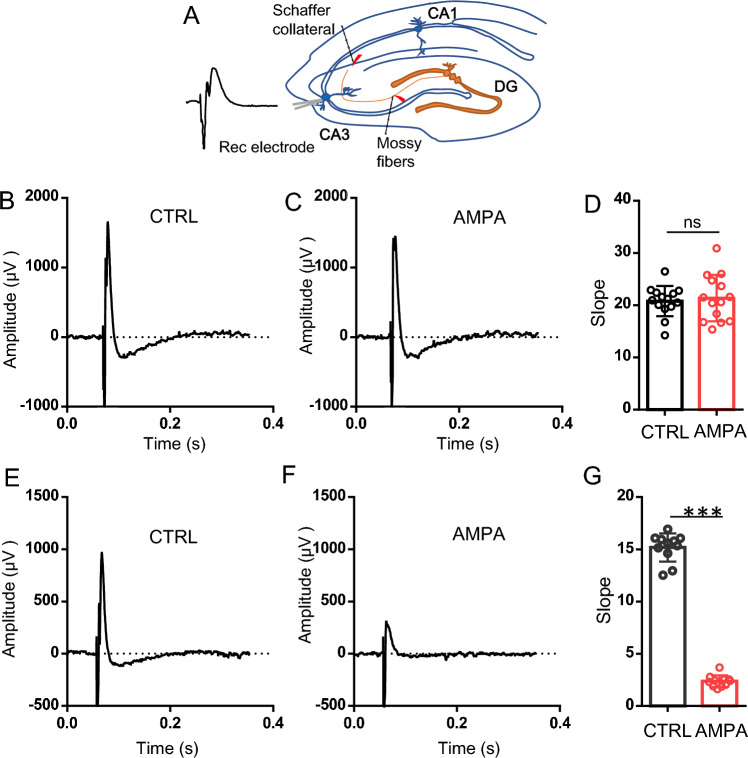
The effect of AMPA on fEPSP in CA3 area of hippocampal slices. (**A**) the schematic illustration about recording of synaptic transmission of CA3 from mossy fibers and recurrent collateral fibers, DG: dentate gyrus, recording electrode was placed at CA3(b) stratum pyramidale; the stimulus electrode was placed at either DG mossy fibers or CA3(c) stratum radiatum. (**B**) and (**C**) Example traces of fEPSP recorded in CA3 stratum pyramidale with stimulus at mossy fibers from dentate gyrus before (**B**) and after AMPA perfusion (**C**). (**D**) The fEPSP slope before and after AMPA perfusion with stimulation of mossy fibers. (**E**) and (**F**) Example traces of fEPSP recorded in CA3 stratum pyramidale with stimulus at CA3 stratum radiatum before (**E**) and after AMPA perfusion (**F**). (**G**) The fEPSP slope before and after AMPA perfusion with stimulation of recurrent collateral fibers.

Previous study demonstrated that perfusion of exogenous NMDA to the hippocampal slice depressed glutamatergic transmission^40^. Consistent with this idea, it is critical to demonstrate the effect of AMPA on the synaptic transmission of area CA3 (mossy fibers and recurrent collateral fibers). It is likely that AMPA-mediated reduction in the oscillatory activity may be due to a depression of glutamatergic transmission in CA3 area. To test this hypothesis, we then recorded the mossy fibers-CA3 synaptic transmission by placing a stimulus electrode in mossy fibers from dentate gyrus (see Fig. 6A) and a record electrode in CA3 stratum pyramidale, and we found that AMPA (5 μM) had no effect on the slope of field excitatory synaptic potential (fEPSP) evoked by mossy fiber stimulation (102.7 ± 8% of Ctrl, vs Ctrl, t_(26)_ = 0.403, *P* = 0.69, n = 14 slices from 6 rats for both Ctrl and AMPA group, Fig. 6A–D). We next recorded the recurrent excitation by placing a stimulus electrode in CA3 stratum radiatum and recording electrode in CA3 pyramidal lawyer and found that AMPA (5 μM) significantly reduced the slope of fEPSP (15.63 ± 4.1% of Ctrl, vs Ctrl, t_(20)_ = 29.132, *P* < 0.001, n = 11 slices from 5 rats for both Ctrl and AMPA group, Fig. 6A,E–G). These data indicate that AMPA downregulation of γ power may be associated with the reduced synaptic transmission of CA3 from recurrent collateral fibers.

## Discussion

AMPARs play a critical role in fast excitatory synaptic transmission and synaptic plasticity^41,42^. Numerous evidence showed multiple neurological disorders are involved in dysregulation of plasticity processes or excitatory synaptic transmission^43^. 

γ oscillations are generated by interaction between excitatory and inhibitory neurons^44^. In this study, we demonstrated that exogenous AMPA inhibited γ power and increased the peak frequency of CCH-induced γ oscillations in the CA3 area of the rat hippocampal slices. AMPA-mediated downregulation of γ power and increase in peak frequency suggests that neurons were able to oscillate at higher frequency given that these neurons no longer need to support oscillation at a higher power.

The frequency of CCH-induced γ oscillations in the mouse hippocampal CA3 was increased by the activation of NMDA receptors (NMDARs) on interneurons^45^. Since NMDAR antagonist had no effect on AMPA mediated γ, it is currently difficult to link a role of NMDAR in AMPA-induced increase in peak frequency.

CCH-induced γ oscillations in the hippocampal CA3 depend on AMPAR-mediated excitation^45^. Hippocampal CA3 area enriched in recurrent collateral which are critical for generation of γ oscillations in CA3. Contrary to the common belief, perfusion of NMDA to the hippocampal slices depressed glutamatergic transmission^40^. In this study, we demonstrated the significant inhibitory effect of exogenous AMPA on the synaptic transmission of area CA3 from recurrent collateral fibers but not from mossy fibers. Thus, the reduction in the oscillatory activity may be the consequence of AMPA-mediated depression of glutamatergic transmission similar to that observed with NMDA in area CA1 of the hippocampus^40^.

Ca^2+^ participates in multiple important physiological activities including network oscillation and synaptic plasticity^46^. We previously demonstrated that L-type calcium channel activation correlated with rapid γ power reduction^47^. In this study, nifedipine had no effect on AMPA downregulation of γ power, suggesting L-type calcium channel was not involved in the role of AMPA.

CP-AMPAR is known to mediate Ca^2+^ influx and CP-AMPAR activation negatively mediated hippocampal γ oscillation, as IEM1460, a selective calcium-permeable AMPA receptor inhibitor enhanced CCH-induced hippocampal γ oscillations^48^. Similarly, CAMKK inhibitor STO-609 also significantly increased γ power. These results indicate that CP-AMPAR/CAMKK signaling may contribute to the AMPA downregulation of γ oscillation.

Either a low concentration of NBQX (1 μM) or IEM1460 had no effect on AMPA-mediated γ reduction, whereas co-administration of IEM1460 and NBQX largely blocked AMPA-mediated γ reduction, indicating the potentiated effect of the combined antagonists on CP-AMPAR and/or CI-AMPAR.

Recently, Pampaloni et al.^49^ identified the slow AMPAR in the hippocampus which provides massive depolarization that can trigger an action potential from a single stimulation and produce short-term potentiation from a purely postsynaptic locus. Whether the slow AMPAR is involved in hippocampal γ oscillations remains to be further determined. Currently we cannot exclude the possibility of slow AMPAR involvement in AMPA-mediated downregulation of γ oscillations.

The rapid downregulation of AMPA on γ oscillation found in this study provides a tool for the rapid, reversible modulation of γ oscillations.γ oscillations are believed to exert a role in cognition and aberrant γ oscillations observed in AD^12^. Studies in APP/PS1 AD mice have shown that prior to significant neuropathological changes, the expression and phosphorylation of CP-AMPAR were increased, which was involved in the regulation of synaptic plasticity at early stages of AD^50^. AD-associated increase in the expressions of CP-AMPAR also linked to abnormal neural network activity^51^. CP-AMPAR inhibition may serve as a strategy for the intervention of neurodegenerative diseases in which calcium homeostasis was disturbed and γ oscillation was impaired. In this study, we demonstrated that CP-AMPAR antagonist IEM1460 and CaMKK inhibitor STO 609 significantly enhanced γ oscillations, IEM1460 and STO 609 alone or in combination may therefore serve as one potentially therapeutic strategy for the AD treatment. Our study provided not only novel insights into the exogenous AMPA-mediated hippocampal γ oscillation but also a strategy for boosting hippocampal γ oscillation, which is critical for the improvement of cognitive function in many neurological disorders.

## Materials and methods

### Experimental animals

Sprague Dawley (SD) rats (male, 4–5 week old) were purchased from Beijing Weitong Lihua Experimental Animal Co., Ltd. The animals were raised in the Key Laboratory of Brain Research of Henan Province. All the experimental methods were performed in compliance with the ARRIVE (Animal Research: Reporting In Vivo Experiments) guidelines. This study was performed following the rules of the International Council for Laboratory Animal Science for the protection of the laboratory animals employed for scientific purposes. The ethics committee for animal study of the Xinxiang Medical University approved the experimental protocol (protocol code: XYLL20210120, date of approval: March 8, 2021). Utmost efforts were made to decrease animal suffering as well as the number of animals utilized in current study.

Prior to the operation, intraperitoneal injection of Sagatal (sodium pentobarbitone, 100 mg kg–1, Rhône Mérieux Ltd., Harlow, United Kingdom) was performed to anesthetize the animals. By the time all pedal reflexes disappeared, the rats were perfused intracardially with oxygenated saturated chilled (5 °C) artificial cerebrospinal fluid (ACSF) in which sodium chloride was replaced with iso-osmotic sucrose. This sucrose-ACSF contained (in mM): 3 KCl, 225 sucrose, 1.25 NaH_2_PO_4_, 24 NaHCO_3_, 0.5 CaCl_2_, 6 MgSO_4_, and 10 glucose (pH: 7.4). The rat brains were then undergone horizontally cutting (400 μm) at 5 °C in the sucrose-ACSF with a Leica VT1000S vibratome (Leica Microsystems, Milton Keynes, United Kingdom). The brain slices were stored in a chamber containing sucrose-ACSF prior to extracellular field recordings.

To record γ oscillation in CA3 of rat hippocampal slices, the slices were transferred to a Haas type interface recording chamber continuously perfused with carbogen (95% O_2_–5% CO_2_) saturated ACSF at room temperature. The recording ACSF contained (in mM): 3 KCl, 126 NaCl, 1.25 NaH_2_PO_4_, 24 NaHCO_3_, 2 CaCl_2_, 2 MgSO_4_, and 10 Glucose (pH: 7.4).

### Electrophysiological recording, data acquisition and analysis

The brain slices were allowed to equilibrate for 1 h in the ACSF saturated with humidified carbogen (95% O^2^ and 5% CO^2^) at 32 °C in the Haas type chamber before recording. Glass microelectrodes containing ACSF (resistance 2–5 MΩ) were employed to record the extracellular field potentials at CA3c stratum pyramidale of rat hippocampus. A NL100AK AC pre-amplifier headstage was used to increase the signal-to-noise ratios before the pulses enter an amplifier (Digitimer Ltd., Welwyn Garden City, United Kingdom). A Neurolog NL106 AC/DC amplifier (Digitimer) was used to amplify field potential and a Neurolog NL125 filter (Digitimer) was utilized for band-pass filter between 0.5 Hz and 2 kHz. The electromagnetic interference originating from the main power supply was eliminated from the extracellular field recordings with one 50 Hz Humbug noise eliminator (Digitimer Ltd.). The extracellular field recording signals were digitized at a sample rate of 5–10 kHz using a CED 1401 plus ADC board (Cambridge Electronic Design, Cambridge, United Kingdom).

The recorded data were analyzed offline with Spike2 software (Cambridge Electronic Design). To provide a quantitative measure of frequency components, power spectra were also plotted using Spike2 software. The power spectra were constructed for 60 s epochs with a fast Fourier transform algorithm. The area under the curve between 20 and 60 Hz was used to quantify the γ power. Autocorrelograms were calculated with Spike2 software using a 500-ms lag from the same local field potential recording employed for γ power calculation. It is generated by fitting the autocorrelation peaks with an exponential function(Y = exp(− Δt/τ)). The decay time constant (τ) of the autocorrelation peaks is utilized to measure the regularity of the oscillation.

### Statistical methods

All statistical tests were performed using SigmaStat software (Sysstat software, San Jose, CA, United States). Where data sets were not different from a normal distribution, results are expressed as mean ± standard error of the mean, between-group comparisons were made with a Student’s t-test and within-group comparisons were made with a paired Student’s *t*-test or a repeated measures ANOVA. Where data sets were different from a normal distribution, results are expressed as median (interquartile range) and between-group differences were tested with a Mann–Whitney U tests. Effects were considered statistically significant if *P* < 0.05. *, **, *** represents *P* < 0.05, 0.01, 0.001, respectively.

### Drugs

CCH was purchased from Sigma. The selective AMPAR agonist AMPA, AMPAR antagonist NBQX, CP-AMPAR antagonist IEM1460, CAMKK inhibitor STO-609, CAMKII inhibitor KN93 and L-type calcium channel blocker nifidepine were purchased from Tocris Bioscience (Bristol, UK). Stock solutions such as AMPA, NBQX, nifidepine, STO-609, KN93, CCH and IEM1460, at thousand times the final concentration, were dissolved in DMSO, and stored in individual aliquots at − 20 °C. Final solutions were prepared freshly on the day of the experiment.

## Data Availability

Data will be available from the corresponding author on request.
